# South Georgia Medical Society

**Published:** 1875-08

**Authors:** 


We learn that the physicians of Thomas and adjoining counties of the southern border of Georgia, held a meeting in Thomasville on the 22d of June last, and formed themselves into a local medical society, under the name of the South Georgia Medical Society. The organization consists of President, T. S. Hopkins, M.D., of Thomas county; Vice-President, J. A. Butts, M.D., of Decatur county; Secretary, D. S. Brandon, M.D., of Thomas county; Treasurer, W. J. Harrell, M.D., of Decatur county. The meetings of the society are to be held quarterly; the next in course being set down for the third Tuesday in September, at Bainbridge, Decatur county.

We are gratified to see this manifestation of professional life on the part of our brethren in the southern border, and trust that the physicians of that region will rally to its support. The beneficial influences to the individuals concerned, as well as to the profession at large and the general community, which grow out of these local medical organizations, are varied and valuable. Such professional re-unions tend to break down the selfishness which isolation naturally engenders; they beget mutual forbearance and respect, personal regard and confidence, friendship and brotherly love. The discussion of scientific subjects brings a comparison of views and practices, alike instructive to those who give as to those who may receive. We think every such organization should have its Reporter, judiciously chosen, whose duty it should be do *condense* the discussions of the body for publication in the medical Journals. Such discussions from the pencil of a skilful reporter, are always read with interest by the medical public; and the fact that the discussions are thus given to the public gives incentive for greater deliberation, precision and care in the discussions.

A necessary concomitant in medical organizations is disciplinary power over the individual members, and herein experience has found an element of strength for self-protection upon the one hand, and an element of weakness and evanescence upon the other. To make such bodies strong and permanent it is necessary that brotherly kindness and charity shall largely prevail. We should eagerly invoke the fairy gift "to see ourselves as others see us;" and we should never forget that it is human to err—that it is christian to forbear—that is godlike to forgive. Let discipline be lodged in the hands of the few chosen for their age, experience, temperance and freedom from bias. Let judgment be wise and just, but sure and summary; above all, let the rod descend in charity and loving kindness.

